# Mapping evidence of mobile health technologies for disease diagnosis and treatment support by health workers in sub-Saharan Africa: a scoping review

**DOI:** 10.1186/s12911-020-01381-x

**Published:** 2021-01-06

**Authors:** Ernest Osei, Desmond Kuupiel, Portia Nelisiwe Vezi, Tivani P. Mashamba-Thompson

**Affiliations:** 1 Second Floor of George Campbell Building, Department of Public Health Medicine, School of Nursing and Public Health, University of KwaZulu-Natal, Howard College Campus, Durban, 4001 South Africa; 2 Research for Sustainable Development Consult, Sunyani, Ghana; 3 Faculty of Health Sciences, University of Pretoria, Pretoria, South Africa

**Keywords:** mHealth technologies, Mobile health, mHealth applications, mHealth apps, Disease diagnosis, Disease screening, Diagnostic accuracy, Treatment support, Therapeutic procedures, Health workers, Sub-Saharan Africa

## Abstract

**Background:**

The rapid growth of mobile technology has given rise to the development of mobile health (mHealth) applications aimed at treating and preventing a wide range of health conditions. However, evidence on the use of mHealth in high disease burdened settings such as sub-Sharan Africa is not clear. Given this, we systematically mapped evidence on mHealth for disease diagnosis and treatment support by health workers in sub-Saharan Africa.

**Methods:**

We conducted a scoping review study guided by the Arksey and O’Malley’s framework, Levac et al. recommendations, and Joanna Briggs Institute guidelines. We thoroughly searched the following databases: MEDLINE and CINAHL with full text via EBSCOhost; PubMed; Science Direct and Google Scholar for relevant articles from the inception of mHealth technology to April 2020. Two reviewers independently screened abstracts and full-text articles using the eligibility criteria as reference. This study employed the mixed methods appraisal tool version 2018 to assess the methodological quality of the included studies.

**Results:**

Out of the 798 articles identified, only 12 published articles presented evidence on the availability and use of mHealth for disease diagnosis and treatment support by health workers in SSA since 2010. Of the 12 studies, four studies were conducted in Kenya; two in Malawi; two in Nigeria; one in South Africa; one in Zimbabwe; one in Mozambique, and one in Lesotho. Out of the 12 studies, one reported the use of mHealth for diseases diagnosis; three reported the use of mHealth to manage HIV; two on the management of HIV/TB; two on the treatment of malaria; one each on the management of hypertension; cervical cancer; and three were not specific on any disease condition. All the 12 included studies underwent methodological quality appraisal with a scored between 70 and 100%.

**Conclusions:**

The study shows that there is limited research on the availability and use of mHealth by health workers for disease diagnosis and treatment support in sub-Saharan Africa. We, therefore, recommend primary studies focusing on the use of mHealth by health workers for disease diagnosis and treatment support in sub-Saharan Africa.

## Background

Globally, there is tremendous growth in mobile technology which has given rise to the development of mobile health (mHealth) technologies for managing a wide range of health conditions [1]. Health-related programmes using mobile communication technologies are evolving to help strengthen healthcare systems and deliveries [2–4]. The Joint United Nations Programme on HIV/AIDS (UNAIDS) and World Health Organization (WHO) have jointly included wireless mobile communication as part of their strategic plans in implementing health policies and programmes [4, 5]. Globally, smartphone users have been estimated to be about 60% according to the Global Speciale for Mobile Association (GSMA) 2018 report, and this is projected to increase to almost 79% by 2025 [6]. In 2018, the estimated number of smartphone users in Sub-Saharan Africa (SSA) was nearly 36%, and this is predicted to rise to about 66% by 2025 [7]. Because of this high mobile phone penetration rate and its uniqueness, it has become a compelling tool for promoting healthcare delivery and bridging the gaps in accessing quality healthcare [8–10]. The mobile phone text-messaging component is not only used for normal global communication, but it is also being utilized to support healthcare delivery and prevent diseases [11].

Mobile health technology can be defined as the use of mobile devices, their various components as well as other related technologies in healthcare delivery [12]. Mobile health is an emerging and promising way to improve disease prevention, diagnosis, treatment compliance, medication adherence, and honouring clinic appointments thereby enhancing health outcomes [13]. Mobile phone technology allows easy remote communication between health workers and their patients in hard-to-reach communities with poor access to healthcare due to deplorable roads or transportation challenges [14–16].

Previous studies have demonstrated that mHealth technologies can improve healthcare outcomes such as health workers adhering to case management standards and guidelines [11, 17–19]. One main application of mHealth intervention that can improve health workers' performance is to send these health workers short message prompts, educational materials on new diagnostic and treatment procedures of cases, clinical guidelines, and others [11, 17, 20, 21]. In SSA, where healthcare systems continuously face problems like inadequate infrastructure, shortage of resources, and an increasing burden of communicable and non-communicable diseases such as HIV, tuberculosis, hypertension, diabetes, among others [22]. To this end, mobile health technology is useful to support disease diagnosis and treatment of such diseases in SSA. Mobile health technology also helps health workers to support their patients and prevent late disease detection, reduce mortalities, improve poor treatment outcomes, and many others [23].

In SSA, people in limited-resource settings may have poor access to quality healthcare due to poor road networks, long-distance travel, lack of trained health professionals, lack of health facilities, among others [16, 23]. In the light of these healthcare challenges and the potentials of mHealth for improving access to healthcare, mHealth could be adopted by health workers and policymakers to support the provision of quality healthcare to people living in hard-to-reach communities. Despite this, no study has mapped literature on mHealth for disease diagnosis and treatment support by health workers in SSA to the best of our knowledge. Hence, there is an urgent need to explore the role of mHealth for disease diagnosis and treatment support by health workers and identify gaps for future research. The focus of this study is using mHealth applications for disease diagnosis, screening, testing, and treatment support by health workers in SSA. Therefore, this present study mapped evidence on mHealth for disease diagnosis and treatment support by health workers in SSA.

## Methods

### Study design

This study is part of a larger study aimed at examining the accessibility of mHealth technologies for disease diagnosis and treatment support by health professionals in Ghana. A scoping review methodology was chosen because it is the most appropriate approach used to synthesize the available body of evidence that has not been reviewed comprehensively. Scoping reviews help to understand research fields that are mostly in early stages since it allows the mapping of key concepts, sources, and types of available evidence that leads to identifying research gaps within the existing literature [24]. This scoping review was guided by the 2005 Arksey and O’Malley’s framework [24], Levac et al. 2010 recommendations [25], and the 2015 Joanna Briggs Institute [26] guidelines. We searched quantitative studies, qualitative studies, mixed-method studies, randomized controlled trials, non-randomized controlled trials, and grey literature that examined the use of mHealth for disease diagnosis and treatment support by health workers in SSA. The results of this review were presented following the Preferred Reporting Items for Systematic Reviews and Meta-analysis: Extension for Scoping Review (PRISMA-ScR) guidelines [27].

## Identifying the research question

Research question: What is the evidence on the availability and use of mHealth for disease diagnosis and treatment support by health workers in SSA?

The Population, Concept, and Context (PCC) framework developed by Joanna Briggs Institute [26] was used to determine the eligibility of our primary research question as illustrated in Table 1.

**Table 1:** PCC framework for defining the eligibility of the studies for the primary research question

Determinants	Description
Population	*Health workers* All categories of trained health professionals such as Nurses, Midwives, Doctors, Physician Assistants/Medical Assistants, Community health workers, Pharmacists/Pharmacy technicians, Biomedical scientists/Laboratory technicians, Radiologists, and several other allied professionals working in healthcare facilities located in sub-Saharan Africa
Concept	*Disease diagnosis and treatment support* *Disease diagnosis* Use of mHealth as diagnostic apps to screen or examine patients to identify or detect any form of disease or disorder *Treatment support* Use of mHealth to provide treatment and guiding patients to manage their disease conditions without their physical presence at the health facility
Context	*Availability and use in sub-Saharan Africa* *Availability* mHealth being accessible, usable, and obtainable upon the demand to perform a required function *Use* Process of employing mHealth to accomplish a task such as a diagnosis, treatment, control/prevention, and management of diseases

### Data sources and literature search

A systematic literature search was conducted from MEDLINE and CINAHL with full text via EBSCOhost; PubMed; Science Direct; and Google Scholar databases. The database searches were from the inception of mobile health technology to July 2019 and an updated search in April 2020 using the following keywords: “mHealth technologies”, “mobile health”, ‘‘mHealth applications’’, ‘‘mHealth apps’’, “disease diagnosis”, ‘‘disease screening’’, ‘‘diagnostic accuracy’’, “treatment support”, ‘‘therapeutic procedures’’, ‘‘health workers’’ and ‘‘sub-Saharan Africa’’ (Additional file 1). Boolean terms (AND/OR) were used to separate our keywords.

Medical subject headings (MeSH) were also used in the electronic database search. Date and language limitations were removed to widen the scope of the search to help capture almost all the full range of literature on mHealth for disease diagnosis and treatment support. The year of publication was from the time mobile health technology was introduced to support healthcare delivery to April 2020 to identify the pattern of reports on mHealth for disease diagnosis and treatment support by health workers in SSA. Reference lists of the included articles were also searched thoroughly to source for relevant literature.

### Study selection

Our study selection was conducted in three stages. At the first stage, E.O conducted the electronic database search and screened titles of articles with guidance from the eligibility criteria. After the title screening, E.O and P.N.V independently screened the abstracts and full articles in parallel. Discrepancies in the reviewers’ responses at the abstract stage were resolved via a discussion until an agreement was reached. A third reviewer, D.K was contacted to resolve the discrepancies between the reviewers’ responses at the full-text screening stage through a discussion.

### Eligibility criteria

#### Inclusion criteria

The following were included:

- Articles that reported evidence of health workers using mHealth.
- Articles that presented evidence on mHealth (text message, voice calls, multimedia messaging, mobile apps, emergency toll-free services, among others).
- Articles that reported evidence on the availability of mHealth for disease diagnosis.
- Articles presenting evidence on the availability of mHealth for treatment support.
- Articles reporting evidence on the use of mHealth for disease diagnosis.
- Articles that reported evidence on the use of mHealth for treatment support.
- Articles presenting evidence from sub-Saharan Africa.

### Exclusion criteria

We excluded the following:

- Articles that reported evidence of patients using mHealth.
- Articles that reported evidence on eHealth.
- Articles that presented evidence on mHealth for surveillance.
- Articles that presented evidence on mHealth for health education.
- Articles that reported evidence on mobile clinics.
- Articles that reported evidence on mHealth for communication.
- Articles that presented evidence on mHealth for data collection.
- Articles that presented evidence on evaluation or assessment of mHealth.
- Articles that presented outside sub-Saharan Africa.

### Data charting

The included selected articles were comprehensively read for data extraction using a standardized data extraction tool. We extracted data on the following: author and year of publication, country of the study, geographical setting (rural/urban/semi-urban), study setting, and study design. Other information such as target population, type of mHealth devices, nature of mHealth intervention, the purpose of mHealth, disease diagnosis, and treatment support were also extracted as shown in Table 2.

**Table 2:** Characteristics of the included articles

Author and date	Country	Geographical setting (rural/urban/semi-urban)	Study setting	Study design	Target population	Aim of the study	Type of mHealth devices	Nature of mHealth intervention	Purpose of mHealth intervention	Treatment support
Kaunda-Khangamwa et al. [17]	Malawi	Urban	Government hospitals and Christian Health Association of Malawi hospitals	Cluster randomized trial study	Health workers	The study aimed to assess the effect of mobile phone text message reminders on health workers’ adherence to case management guidelines for malaria and other diseases	Mobile phones	Short messaging service (SMS)	To assist health workers to adhere to patients’ case management in malaria, pneumonia, and diarrhoea treatment	Reminders
Larissa et al. [31]	Kenya	Urban	Kendu Bay and Rachuonyo districts hospitals	Descriptive qualitative study	Community health workers	The study aimed to examine what specific content and forms of mobile communication are acceptable to support the prevention of mother-to-child transmission (PMTCT)	Mobile phones	SMS and voice calls	To help infected HIV pregnant mothers to adhere to ART medication procedure to improve PMTCT	Reminders
Marufu et al. [34]	Zimbabwe	Urban	District hospital	Quantitative study	Health workers	The study aimed to determine the use of mHealth and identifying and describing the opportunities and the challenges faced by medical doctors in using mHealth at a specific health care facility	Cell phones	Voice calls and mobile website	Voice calls were used for appointments and medication reminders Mobile website for medical/clinical research	Reminders
Moodley et al. [33]	South Africa	Urban	Cape Town tertiary hospital	Mixed method study	Primary health workers	The study aimed to determine the feasibility of mobile health technology to improve management and follow-up of clients with cervical cancer precursor lesions	Mobile phones	SMS	For delivering test results in cervical cancer and appointment reminders	Reminders and follow-ups
Nelissen et al. [32]	Nigeria	Urban	University teaching hospital and Pharmacy-based care model	Mixed method study	Health workers	The study aimed to investigate the feasibility of a pharmacy-based hypertension care model using mHealth	Mobile phones	Mobile apps and phone calls	For monitoring and ensuring that patients adhere to medication procedures of hypertension	Reminders and follow-ups
Nhavoto et al. [1]	Mozambique	Semi-urban	Machava II health centre, Matolla I & II health centres, Namaacha health centre, and Ndlavela Health centre	Qualitative study	Health workers	The study aimed to examine patients' and healthcare workers' views on an mHealth intervention aiming to support retention in antiretroviral therapy (ART) and tuberculosis (TB) treatment	Mobile phones	SMS	For appointments, collection of antiretroviral therapy (ART) and tuberculosis drugs, and medication adherence	Reminders and follow-ups
Smillie et al. [30]	Kenya	Semi-urban	Kibera Community health centre (KCHC) and Research Foundation (AMREF) clinic	Qualitative study	Community health workers	The study aimed to explore the experiences of people living with HIV and their perceptions of communicating via text message with healthcare providers	Mobile phones	SMS (WelTel) and voice calls	To monitor HIV patients’ conditions and provide medication guidelines	Reminders and follow-ups
Lester et al. [4]	Kenya	Urban, semi-urban and rural	University of Nairobi Pumwani clinic, Coptic Hope Centre for Infectious Diseases and Kajiado clinic	Randomized controlled trial study	Health workers	The study aimed to assess whether mobile phone communication between health workers and patients starting ART improved drug adherence and suppression of plasma HIV1	Mobile phones	SMS	To assist HIV diagnosed patients to adhere to treatment compliance and medication adherence	Reminders and follow-ups
Hirsch-Moverman et al. [14]	Lesotho	Semi-urban	Berea district health facilities	Cluster randomized controlled trial study	Health workers	The study aimed to describe the use and acceptability of mHealth by patients with HIV/TB and health care providers	Mobile phones	SMS	For clinic appointments and support patients on HIV and TB medication adherence	Reminders and follow-ups
Zurovac et al. [11]	Kenya	Rural	Rural government health centres	Cluster randomized controlled trial study	Health workers	This study aimed to assess whether text-message reminders sent to health workers’ mobile phones could improve and maintain their adherence to treatment guidelines for outpatient paediatric malaria	Mobile phones	SMS	To assist health workers to adhere to malaria treatment guidelines	Reminders
Hardy et al. [36]	Malawi	Rural	Village clinics within Mzimba & Rumphi districts	Cluster randomized controlled trial study	Community health workers	To evaluate the added value of the Supporting LIFE electronic Community Case Management Application (SL eCCM App) compared to paper-based Community Case Management on urgent referral, re-consultation, and hospitalization rates, in two districts in Northern Malawi	Mobile phones	Mobile apps	To help community health workers to adhere to referral recommendations or guidelines in treating sick children under 5-years	Reminders and follow-ups
Yahya., 2019 (37)	Nigeria	Urban	Public, private and faith-based hospitals	Quantitative study	Health workers	To examine ownership, frequency and pattern of use and problems encountered in the use of smartphones among all category of medical doctors in hospitals in Kaduna, Nigeria	Smart-phones	Mobile apps	To assist medical doctors in checking details of diseases, making differential diagnoses of diseases, determining drugs that might be useful for a particular condition, and assessing their interactions with other drugs	Reminders

### Quality assessment of the included studies

We used the mixed method appraisal tool **(**MMAT) version 2018 [28] for methodological appraisal of all included primary studies. The included primary articles were appraised using the appropriate study designs as stipulated by the MMAT. The percentage quality score of all the included primary articles was then calculated for each and interpreted as ≤ 50%-low quality, 51–75% -average quality, and 76–100%-high quality [29].

### Collating, summarizing, and reporting

This review study employed a thematic analysis to present the findings from the existing literature. Our narrative literature was then structured around the themes derived from the study results or outcomes. The themes that emerged from the articles were: availability of mHealth, use of mHealth in terms of treatment, prevention and management of HIV, TB, hypertension, cancer, malaria, pneumonia and diarrhoea conditions, use of mHealth for disease diagnosis, and acceptability of mHealth.

### Screening results

**Fig. 1:**
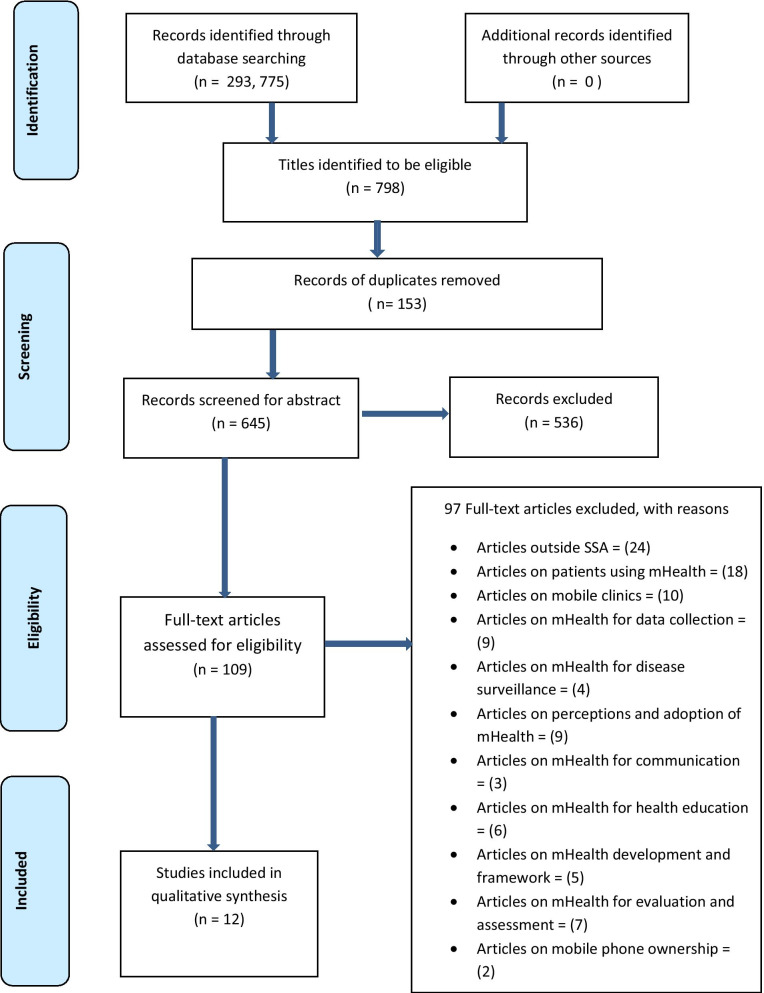
PRISMA-ScR flow chart showing literature search and selection of studies

Out of the 293, 775 articles produced by this scoping review from the database searches, 798 articles met the eligibility criteria following the title screening. Of these 798 articles, 153 duplicates were removed leaving 645 articles eligible for abstract screening. A total of 536 articles were excluded following abstracts screening, and 109 were found eligible for full article screening. Subsequently, at the full article screening stage, 97 articles were excluded as shown in Fig. 1 which demonstrates the PRISMA-ScR flow chart of literature search and selection of studies. In all, a total of 12 articles met our eligibility criteria for the extraction of data from the initial and updated searches. Following full article screening, there was a 91.74% agreement versus 74.27% expected by chance which constitutes a high degree of agreement (Kappa statistics = 0.68, p value < 0.05). In addition, McNemar's chi-square statistic suggests that there is not a statistically significant difference in the proportions of yes/no answers by the reviewers, with a p value > 0.05 (Additional file 2).

### Characteristics of included articles

**Fig. 2:**
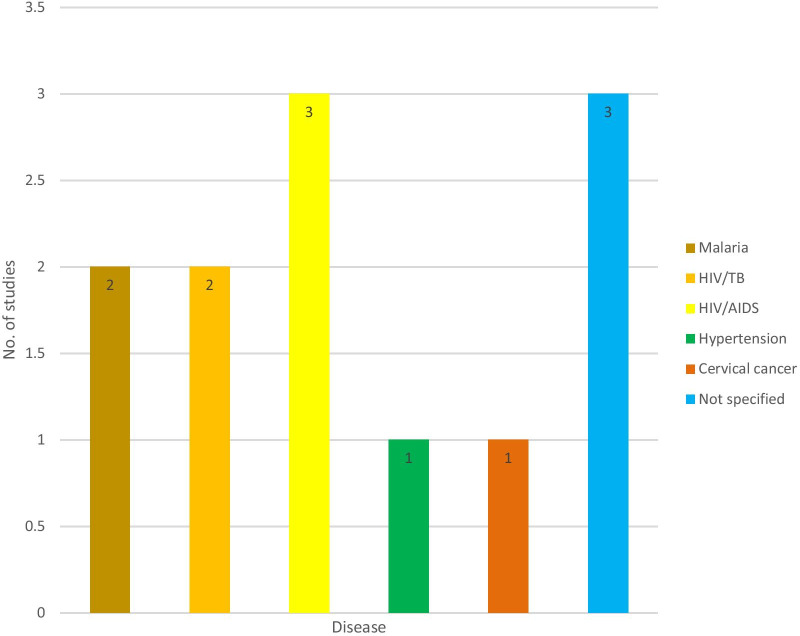
Distribution of included studies by diseases

Out of the 12 included studies, only one study reported on the availability and use of mHealth for disease diagnosis (132); three studies reported on the availability and use of mHealth for treatment support on HIV/AIDS [4, 30, 31]; two on HIV and TB [1, 14]; two on malaria, pneumonia and diarrhoea support [11, 17]; one on hypertension [32]; one on cervical cancer support [33] and three studies did not specify any disease [34–36]. Figure 2 shows the distribution of included studies by diseases. The characteristics of the included articles are summarised in Table 2. The 12 included articles comprised three qualitative studies [1, 30, 31], five cluster randomized controlled trials [4, 11, 14, 17, 36], two quantitative studies [34, 35], and two mixed-method studies [32, 33]. All the included articles were published in the English language from 2010 to 2019.

**Fig. 3:**
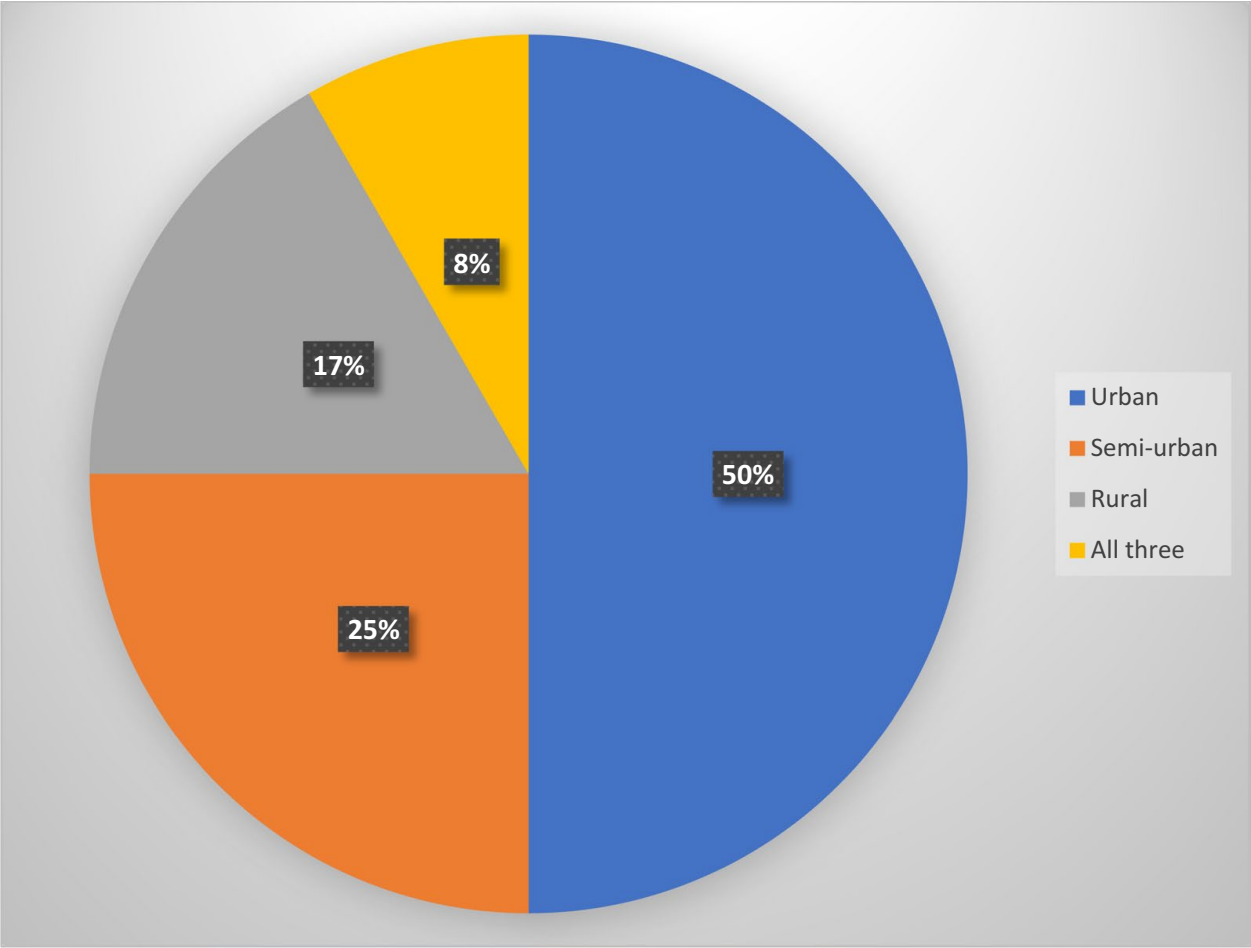
Distribution of included studies by geographical settings

All the included studies presented evidence on mHealth for treatment support by health workers in SSA. Out of the 12 included primary studies, only one study reported evidence on mHealth for disease diagnosis by health workers in SSA. In terms of geographical settings, six of the 12 includes studies were conducted in urban settings [17, 31–35]; three studies were conducted in semi-urban settings [1, 14, 30]; two studies were conducted in rural settings [11, 36], and only one study was conducted in rural, semi-urban and urban settings [4]. Figure 3 shows the distribution of included studies by geographical settings. Of the 12 included studies, four studies were conducted in Kenya [4, 11, 30, 31]; two in Malawi [17, 36]; two in Nigeria [32, 35]; one in Zimbabwe [34]; one in South Africa [33]; one in Mozambique [1] and one in Lesotho [14].

### Quality of the evidence

All the 12 included primary studies underwent methodological quality assessment using the 2018 version of MMAT [28]. The 12 included primary studies that underwent the methodological quality appraisal scored within the range of 70 and 100%. Out of the 12 included primary studies, eight studies scored 100% which is the highest quality score [1, 17, 30–35]; two studies had an average of 85.7% quality score [4, 36]; and the two remaining studies also had the lowest quality score of 71.4% [11, 14] (Additional file 3).

## Summary of study findings

**Fig. 4:**
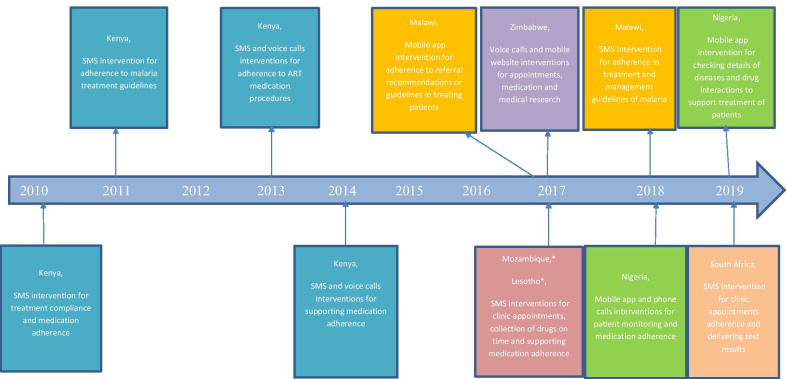
Distribution of studies with countries of publication and the type of mHealth intervention

All the included primary studies presented evidence on the availability and use of mHealth for treatment support by health workers in SSA. However, only one study reported evidence on the availability and use of mHealth for disease diagnosis by health workers in SSA. Figure 4 demonstrates the time mHealth for treatment support was first published, countries with mHealth, type of mHealth interventions used, and the purpose of the mHealth interventions. The following are the themes that emerged from the included studies: availability of mHealth, use of mHealth in terms of treatment, prevention and management of HIV, TB, hypertension, cancer, malaria, pneumonia and diarrhoea conditions, use of mHealth for disease diagnosis, and acceptability of mHealth.

### Availability of mHealth

All the included studies reported on the availability of mHealth [1, 4, 11, 14, 17, 30–36]. Studies conducted in Kenya reported on the availability of mHealth to support pregnant women living with HIV to comply with antiretroviral drugs medication and improve the prevention of mother-to-child-transmission [31]; help HIV/AIDS patients adhere to medication procedures [4, 30]; and encourage health professionals to adhere to standard guidelines when treating malaria conditions [11]. A study in Mozambique indicated the availability of mHealth for supporting HIV and TB patients to honour their clinical appointments and collect their drugs on time [1].

The availability of mHealth was also reported in Lesotho where mHealth was used to help patients reduced the number of missed clinical appointments and collected their drugs on time [14]. Kaunda-Khangamwa et al. [17] demonstrated the availability of mHealth to encourage health workers to strictly adhere to standard guidelines in the treatment of malaria, pneumonia, and diarrhoea cases. Nelissen et al. [32] conducted a mixed-method study that reported on the availability of mHealth to help hypertension patients adhere to or comply with treatment procedures. Moodley et al. [33] reported evidence on the availability of mHealth for supporting patients to manage their cancer conditions.

Marufu et al. [34] conducted a quantitative study that revealed the availability of mHealth to set-up clinical appointments, remote consultation, and adherence to medication procedures. Yahya presented evidence on the availability of mHealth for checking guidelines about a disease condition, determining when a drug might be useful for a particular disease condition, and assessing their interactions with other drugs to support the treatment of patients [35]. A study conducted in Malawi found the availability of mHealth for supporting community health workers to adhere to referral recommendations in the treatment procedures of patients [36]. We found that mHealth for treatment support is available in only seven SSA countries with few diseases which demonstrates a major gap in literature.

### Use of mHealth for treatment support

All the included studies reported on the use of mHealth technology for treatment support [1, 4, 11, 14, 17, 30–36]. Nelissen et al. [32] showed that both healthcare providers and patients perceived the mHealth app as supportive and attractive in managing patients’ illnesses. Zurovac et al. [11] suggested that the text messages helped health workers to improve their quality of disease management skills. Their study results further suggested that the proper management of artemether-lumefantrine medications improved the treatment and cure of children suffering from malaria [11].

A similar study conducted in Malawi revealed that the SMS intervention assisted health workers to comply with standards and guidelines in treating malaria cases, and improved their case management skills [17]. The results of this study further showed that some clinicians used the message received as reference materials to support the treatment and cure of such diseases [17]. Another study conducted in Malawi showed that mobile app helped community health workers to strictly comply with referral guidelines in treating severely sick children under 5 years [36].

Other studies conducted in Kenya also reported that mHealth interventions provided by health workers assisted HIV patients to comply with treatment procedures and stick to medication adherence [4, 30, 31]. Also, studies conducted in Mozambique and Lesotho showed that the SMS intervention provided by health workers helped HIV/TB patients to stay in treatment and increased treatment compliance [1, 14]. The findings presented evidence on the use of mHealth for only malaria, pneumonia, and diarrhoea conditions demonstrating a gap as mHealth could be used to support the treatment of several other diseases in SSA.

#### Use of mHealth for prevention of mother-to-child transmission of HIV

Only one study reported on the use of mHealth intervention to prevent the transmission of diseases [31]. Larissa et al. [31] conducted a study aimed at examining what specific content and forms of mobile communication are acceptable to support the prevention of mother-to-child transmission. The results revealed that SMS reminders sent by health workers assisted HIV-infected pregnant women to stick to the prescribed antiretroviral medication procedures to prevent their unborn babies from contracting the virus [31]. The results also indicated that HIV-infected pregnant women had remote access to their healthcare providers and could easily request more antiretroviral drugs via mHealth without travelling to the health facility [31]. The findings presented evidence on the use of mHealth to prevent the transmission of only HIV condition but no evidence on the use of this intervention to prevent the transmission of other infectious and non-infectious diseases in SSA.

#### Use of mHealth for management of diseases

Six studies reported on the use of mHealth for the management of disease conditions like hypertension, cancer, HIV, and tuberculosis (TB) [1, 4, 14, 30, 32, 33]. Nhavoto et al. [1] suggested that mHealth reminders provided by health workers assisted HIV and TB patients to comply with treatment procedures, collection of drugs on time, and adhering to clinical appointments. A randomized controlled trial study carried out by Lester et al. [4] demonstrated that patients who received the SMS intervention adhered to ART medication procedures. The results also showed that the SMS intervention helped to reduce viral replication via ART, hence decreasing the transmission of HIV1 to new partners [4].

Hirsch-Moverman et al. [14] also conducted a similar study which suggested that patients perceived their adherence to clinic appointments and medication procedures were due to the SMS received from health workers. Their study results further revealed that mHealth can support patients to have remote access to their healthcare providers to report a side effect, seek advice, or inform them about potential delays in-clinic appointments [14]. Moodley et al. [33] illustrated that SMS sent to patients by health workers improved the management of cervical cancer conditions and encouraged patients’ adherence to clinical colposcopy appointments.

Nelissen et al. [32] revealed that patients’ medication adherence and treatment compliance improved significantly due to regular monitoring by health professionals using the mobile app intervention. Smillie et. al showed that the SMS and voice call reminders helped HIV patients to adhere to the antiretroviral treatment process, medication procedures as well as honouring their clinical appointments [30]. The results of their study further indicated that this intervention helped health workers to better manage HIV cases [30]. We found that mHealth was used to manage only a few communicable diseases (HIV, TB) and non-communicable diseases ( hypertension, cancer), however, a gap was revealed as mHealth could be used to manage many other communicable and non-communicable diseases.

### Use of mHealth for disease diagnosis

Only one study reported evidence on the use of mHealth for disease diagnosis by health workers in SSA [35]. Yahya Husain conducted a quantitative study aimed at examining ownership, frequency, and pattern of use and problems encountered in the use of smartphones among all categories of medical doctors in hospitals in Kaduna, Nigeria [35]. The results indicated that medical doctors used the mobile health app to support disease diagnosis to enhance the quality of healthcare delivery [35]. The results further revealed that these medical doctors also used the mobile app to make differential diagnoses of diseases to make correct decisions of cases to help improve the provision of healthcare services [35]. The findings revealed that only one country reported evidence on the use of mHealth for disease diagnosis which demonstrates a major gap in the literature as mHealth could be used for disease diagnosis in several sub-Saharan Africa countries.

### Acceptability of mHealth

All the twelve included primary studies reported on the acceptability of mHealth by health workers to support quality healthcare delivery [1, 4, 14, 17, 30–36]. Four studies reported that health workers accepted mHealth intervention as a supportive tool to help them improve their clinical management skills in treating disease conditions [11, 17, 35, 36]. The results of these studies also demonstrated that clinicians were appreciative of SMS reminders as reference materials to support the treatment of patients suffering from malaria and other diseases [11, 17]. Three studies also reported that health professionals expressed interest in using mHealth for clinic appointments; remote consultation; medication reminders and delivery of patients' test results [31, 33, 34]. Their results again found that medical doctors accepted mHealth as a medium to help them improve service delivery [31, 33, 34].

Five studies reported that health workers were excited to use text messages to help patients to reduce the number of missed appointments; improve the collection of drugs on time and comply with dosage instructions [1, 4, 14, 30, 32]. One study reported that clinicians accepted mHealth applications for diagnosing diseases and even making a differential diagnosis to guide them to make correct decisions in administering healthcare to their patients [35]. Although health workers generally accepted mHealth applications in healthcare delivery, other studies also reported that some sections of health workers raised issues of confidentiality and privacy breaches which should be of public concern [1, 30, 33]. The findings illustrated that there is limited evidence on the acceptability of mHealth for disease diagnosis and treatment support of some disease conditions in SSA.

## Discussion

This scoping review mapped existing literature on the availability and use of mHealth for disease diagnosis and treatment support by health workers in SSA. The findings illustrated that there is limited published research on the availability and use of mHealth for disease diagnosis and treatment support by health workers SSA. This is of great concern and requires immediate action from all relevant stakeholders as SSA seeks to reduce the high disease burdens and improves poor access to healthcare. The results further demonstrated limited research on the use of mHealth interventions to manage some chronic disease conditions such as HIV, TB, cancer, and hypertension in SSA. Nonetheless, the results demonstrated that these interventions enhanced adherence to treatment and medication procedures, promoted clinical appointment compliance, and improved the collection of drugs on time.

The results also revealed that Kenya had the highest evidence on the use of mHealth to support the management of HIV compared to other sub-Saharan African countries. This potentially could help Kenya to improve healthcare access to the majority of her population under the universal health coverage policy [38]. The review findings also indicated that only one study presented evidence on the use of mHealth for disease diagnosis by health workers in SSA. In spite of this, the results showed that mHealth interventions assisted health workers in making differential diagnoses of diseases to ensure those correct decisions are made on patients’ conditions.

Our review study is partly consistent with other studies carried out in some high-income countries (HICs) [39–42] which found the use of mHealth to manage chronic disease conditions like HIV, TB, hypertension, and cancer. Similar studies conducted in low-and middle-income countries (LMICs) also agree with the findings of this review study which found the use of mHealth improved medication adherence and treatment compliance [43, 44]. Other studies conducted in HICs demonstrated a relatively higher level of research on the use of mHealth to support the treatment procedures of many communicable and non-communicable diseases [40, 44–47] which are at variance with this study.

Roesler et al. and Ochalek et al. conducted studies in Brazil and the US which showed a limited level of research on the use of mHealth to prevent transmission of diseases from one individual to another [48, 49] which are similar to our findings. Also, some studies conducted in HICs and other LMICs indicated a limited level of research on the use of mHealth interventions for diagnostic purposes [50, 51] which are consistent with our study findings. Furthermore, there is a higher level of research from some studies conducted in HICs and other LMICs which showed that health workers used tablets, smartphones, personal digital assistants, handheld devices, and mobile phones to support disease diagnosis and treatment procedures [39–42, 44, 45, 47, 49–51] which are not consistent with this study findings.

The findings demonstrated that only one study reported evidence on the use of mHealth interventions by health workers for disease diagnosis. This finding is worrying and requires redress considering that WHO is advocating for improving access to healthcare, particularly in resource-limited settings [38]. Rural communities in SSA most often have poor roads, lack of transportation, inadequate health infrastructure, poor access to healthcare, among others. Therefore, we recommend more mHealth interventions could be implemented to support disease diagnosis, screening, and testing, particularly in rural communities. This will facilitate early detection and treatment of most diseases and improve health outcomes among the general population in SSA.

The limited level of published research on the use of mHealth by health workers to manage communicable diseases like HIV, TB, and malaria conditions demonstrates that much effort is still required by SSA towards the achievement of the Sustainable Development Goal (SDG) 3.3 [52] target which advocates that by 2030 the epidemics of HIV, TB, malaria, and other communicable diseases will be ended. This is not good and requires urgent attention from all the appropriate stakeholders to initiate new mHealth interventions and/or scale-up existing ones to manage several other communicable diseases in SSA.

Again, the findings showed limited research on the use of mHealth by health workers to manage non-communicable diseases (NCDs) in SSA. This demonstrates that SSA has to do more to achieve the SDG 3.4 [52] target which stipulates that by 2030 pre-mature deaths from NCDs will be reduced by one-third via treatment and prevention. This is worrying and needs an urgent redress if SSA wants to achieve this SDG target. To this end, we propose that more mHealth interventions could be rolled out to support the management of many communicable and non-communicable diseases in SSA to enhance the quality of health outcomes.

### Implications for practice

Majority of the studies were conducted in urban settings where access to quality healthcare is mostly available with modern health facilities and highly skilled health workers. Only a few were conducted in semi-urban and rural settings where access to healthcare or health infrastructure is poorly developed with either no or insufficiently trained health workers. This demonstrates that people in hard-to-reach communities may have to travel long distances with transportation challenges to access healthcare services. This may prevent such people from accessing healthcare which could lead to late disease detection, late detection of drug resistance, poor treatment outcomes, and an increase in health-related mortalities. The study findings also revealed that in SSA, health workers used only mobile phones with text message reminders to support treatment procedures. This may affect many patients in resource-limited settings who cannot read and write and may not benefit fully from this intervention, hence more phone/voice calls intervention should be encouraged.

### Strengths and limitations

Our review included studies conducted in different settings (urban, semi-urban, and rural) which provides an overview of mHealth for disease diagnosis and treatment support by health workers in SSA. This scoping review study to the best of our knowledge is the first comprehensive study to explore the available evidence in literature on the availability and use of mHealth for disease diagnosis and treatment support by health workers in SSA. This study illustrated a substantial gap in literature on the availability and use of mHealth for disease diagnosis by health workers to guide future research in SSA. The methodology of our review study also allowed us to include different study designs; identifying all relevant articles systematically; data charting and analyzing the various study outcomes [24, 53] which may not be performed in review articles.

One important strength of our review study was the removal of limitations of date and language. A comprehensive search for available literature used in this review study is another important strength of the study. Despite all these, it is highly possible that research on mHealth for disease diagnosis and treatment support by health workers in SSA probably existed under different terminologies that were not captured in our study. Nonetheless, we included the Medical Subject Heading terms to capture all relevant available literature. This review study was also limited to studies conducted within sub-Saharan Africa and may not be generalized.

### Recommendations for future research

The review study found that there is limited published research on the availability and use of mHealth interventions by health workers for disease diagnosis in SSA. We recommend future research aimed at exploring the availability and use of mHealth by health workers for disease diagnosis in SSA. The findings further illustrate that mHealth intervention for diagnostic purposes is available in only one country in SSA. To this end, we recommend the implementation of mHealth interventions in many countries in this sub-region to support diagnostic procedures of all kinds of diseases.

Our reviewed study findings also demonstrate that there is limited published research on the availability and use of mHealth for treatment support by health workers in SSA. We, therefore, recommend that more primary studies should be conducted in this setting to examine the availability and use of mHealth to support treatment procedures. The findings also show that mHealth for treatment support is available in only a few SSA countries. We, therefore, recommend the implementation of mHealth interventions in many countries within SSA to support treatment procedures of diseases.

The findings further found that mHealth interventions are mostly found in urban settings in SSA. Hence, we recommend that more primary research should be conducted at primary healthcare clinics to assess the availability and use of mHealth for disease diagnosis and treatment support by health workers. The results also indicate that mobile phone text message reminders as the most commonly used mHealth technology by health workers in SSA. We also recommend that more other forms of mHealth devices like personal digital assistants, tablets, smartphones, and other wearables with more phone/voice calls, multimedia messaging services, and other interventions should be encouraged. Randomized controlled trial studies on mHealth for disease diagnosis and treatment support are also recommended because they are the most appropriate study design for assessing the impact and cost-effectiveness of an intervention.

## Conclusion

The study shows that there is limited research on the availability and use of mHealth by health workers for disease diagnosis and treatment support in SSA. The study demonstrates that mHealth interventions could be used to treat and manage both communicable and non-communicable diseases effectively in SSA. This study, in addition, reveals the overall acceptance of the use of mHealth by health workers to support diagnostic and treatment procedures in SSA. Therefore, we recommend that more primary studies should be conducted in this sub-region on the use of mHealth by health workers for disease diagnosis and treatment support to enhance quality healthcare delivery.

## Supplementary Information

**Additional file 1: **Electronic databases search results for the title screening.

**Additional file 2:** Full articles screening results and output of degree of agreement in Stata version 13.

**Additional file 3:** Methodological quality assessment.

## Data Availability

The data supporting the conclusions of this paper are available through the detailed reference list. No original datasets are presented because this is a review of already existing literature.

## Abbreviations

AIDS: Acquired immunodeficiency syndrome
GSMA: Global Speciale for Mobile Association
HICs: High-income countries
HIV: Human immunodeficiency virus
LMICs: Low-and middle-income countries
MMAT: Mixed method appraisal tool
NCDs: Non-communicable diseases
PCC: Population, concept, and context
PRISMA-ScR: Preferred reporting items for systematic reviews and meta-analysis: extension for scoping review
SDG: Sustainable development goal
SMS: Short messaging service
SSA: Sub-Saharan Africa
TB: Tuberculosis
UNAIDS: Joint United Nations Programme on HIV/AIDS
WHO: World Health Organization
