# Supplementary material for: Mapping evidence of mobile health technologies for disease diagnosis and treatment support by health workers in sub-Saharan Africa: a scoping review
Source: BMC Med Inform Decis Mak. 2021 Jan 6;21:11. doi: 10.1186/s12911-020-01381-x (PMC7789784; doi:10.1186/s12911-020-01381-x)
Supplement: Supplementary file 1 — Additional file 1: Electronic databases search results for the title screening. [file 12911_2020_1381_MOESM1_ESM.docx]

**Additional file 1**: Results from the initial database search

| Dates of Search | Search Engine used | Keywords Search | Retrieved articles | Eligible articles |
| --- | --- | --- | --- | --- |
| 15 July 2019 | PubMed | ((((((((((("telemedicine"[MeSH Terms] OR "telemedicine"[All Fields] OR "mhealth"[All Fields]) AND ("technology"[MeSH Terms] OR "technology"[All Fields] OR "technologies"[All Fields])) OR ("telemedicine"[MeSH Terms] OR "telemedicine"[All Fields] OR ("mobile"[All Fields] AND "health"[All Fields]) OR "mobile health"[All Fields])) OR (("telemedicine"[MeSH Terms] OR "telemedicine"[All Fields] OR "mhealth"[All Fields]) AND applications[All Fields])) OR (("telemedicine"[MeSH Terms] OR "telemedicine"[All Fields] OR "mhealth"[All Fields]) AND ("Appl Plant Sci"[Journal] OR "apps"[All Fields]))) AND (("disease"[MeSH Terms] OR "disease"[All Fields]) AND ("diagnosis"[Subheading] OR "diagnosis"[All Fields] OR "diagnosis"[MeSH Terms]))) OR (("disease"[MeSH Terms] OR "disease"[All Fields]) AND ("diagnosis"[Subheading] OR "diagnosis"[All Fields] OR "screening"[All Fields] OR "mass screening"[MeSH Terms] OR ("mass"[All Fields] AND "screening"[All Fields]) OR "mass screening"[All Fields] OR "screening"[All Fields] OR "early detection of cancer"[MeSH Terms] OR ("early"[All Fields] AND "detection"[All Fields] AND "cancer"[All Fields]) OR "early detection of cancer"[All Fields]))) AND (("diagnosis"[MeSH Terms] OR "diagnosis"[All Fields] OR "diagnostic"[All Fields]) AND accuracy[All Fields])) AND (("therapy"[Subheading] OR "therapy"[All Fields] OR "treatment"[All Fields] OR "therapeutics"[MeSH Terms] OR "therapeutics"[All Fields]) AND support[All Fields])) OR ("therapeutics"[MeSH Terms] OR "therapeutics"[All Fields] OR ("therapeutic"[All Fields] AND "procedures"[All Fields]) OR "therapeutic procedures"[All Fields])) AND (("health"[MeSH Terms] OR "health"[All Fields]) AND ("occupational groups"[MeSH Terms] OR ("occupational"[All Fields] AND "groups"[All Fields]) OR "occupational groups"[All Fields] OR "workers"[All Fields]))) AND ("africa south of the sahara"[MeSH Terms] OR ("africa"[All Fields] AND "south"[All Fields] AND "sahara"[All Fields]) OR "africa south of the sahara"[All Fields] OR ("sub"[All Fields] AND "saharan"[All Fields] AND "africa"[All Fields]) OR "sub saharan africa"[All Fields]) AND ("1900/01/01"[PDAT] : "2019/07/15"[PDAT]) | 3,246 | 143 |
| 15 July 2019 | Science Direct | mobile health OR mHealth applications OR mHealth apps AND disease diagnosis AND diagnostic accuracy AND treatment support AND health workers AND sub-Saharan Africa | 205,280 | 220 |
| 16 July 2019 | Google Scholar | mobile health OR mHealth applications OR mHealth apps AND disease diagnosis OR disease screening AND diagnostic accuracy AND treatment support OR therapeutic procedures AND health workers AND sub-Saharan Africa | 4,350 | 184 |
| 16 July 2019 | EBSCOhost (MEDLINE and CINAHL with full text) | mHealth technologies OR mobile health OR mHealth applications OR mHealth apps AND disease diagnosis OR disease screening AND diagnostic accuracy AND treatment support OR therapeutic procedures AND health workers AND sub-Saharan Africa | 42, 647 | 204 |
| **Additional database searches** | | | | |
| Dates of Search | Search Engine used | Keywords search | Retrieved articles | Eligible articles |
| 23 April 2020 | PubMed | ((((((((((("telemedicine"[MeSH Terms] OR "telemedicine"[All Fields] OR "mhealth"[All Fields]) AND ("technology"[MeSH Terms] OR "technology"[All Fields] OR "technologies"[All Fields])) OR ("telemedicine"[MeSH Terms] OR "telemedicine"[All Fields] OR ("mobile"[All Fields] AND "health"[All Fields]) OR "mobile health"[All Fields])) OR (("telemedicine"[MeSH Terms] OR "telemedicine"[All Fields] OR "mhealth"[All Fields]) AND applications[All Fields])) OR (("telemedicine"[MeSH Terms] OR "telemedicine"[All Fields] OR "mhealth"[All Fields]) AND ("Appl Plant Sci"[Journal] OR "apps"[All Fields]))) AND (("disease"[MeSH Terms] OR "disease"[All Fields]) AND ("diagnosis"[Subheading] OR "diagnosis"[All Fields] OR "diagnosis"[MeSH Terms]))) OR (("disease"[MeSH Terms] OR "disease"[All Fields]) AND ("diagnosis"[Subheading] OR "diagnosis"[All Fields] OR "screening"[All Fields] OR "mass screening"[MeSH Terms] OR ("mass"[All Fields] AND "screening"[All Fields]) OR "mass screening"[All Fields] OR "screening"[All Fields] OR "early detection of cancer"[MeSH Terms] OR ("early"[All Fields] AND "detection"[All Fields] AND "cancer"[All Fields]) OR "early detection of cancer"[All Fields]))) AND (("diagnosis"[MeSH Terms] OR "diagnosis"[All Fields] OR "diagnostic"[All Fields]) AND accuracy[All Fields])) AND (("therapy"[Subheading] OR "therapy"[All Fields] OR "treatment"[All Fields] OR "therapeutics"[MeSH Terms] OR "therapeutics"[All Fields]) AND support[All Fields])) OR ("therapeutics"[MeSH Terms] OR "therapeutics"[All Fields] OR ("therapeutic"[All Fields] AND "procedures"[All Fields]) OR "therapeutic procedures"[All Fields])) AND (("health"[MeSH Terms] OR "health"[All Fields]) AND ("occupational groups"[MeSH Terms] OR ("occupational"[All Fields] AND "groups"[All Fields]) OR "occupational groups"[All Fields] OR "workers"[All Fields]))) AND ("africa south of the sahara"[MeSH Terms] OR ("africa"[All Fields] AND "south"[All Fields] AND "sahara"[All Fields]) OR "africa south of the sahara"[All Fields] OR ("sub"[All Fields] AND "saharan"[All Fields] AND "africa"[All Fields]) OR "sub saharan africa"[All Fields]) AND ("2019/07/16"[PDAT] : "2020/04/23"[PDAT]) | 151 | 07 |
| 23 April 2020 | Google scholar | mobile health OR mHealth applications OR mHealth apps AND disease diagnosis OR disease screening AND diagnostic accuracy AND treatment support OR therapeutic procedures AND health workers AND sub-Saharan Africa | 869 | 11 |
| 24 April 2020 | EBSCOhost (MEDLINE and CINAHL with full text | mHealth technologies OR mobile health OR mHealth applications OR mHealth apps AND disease diagnosis OR disease screening AND diagnostic accuracy AND treatment support OR therapeutic procedures AND health workers AND sub-Saharan Africa | 3,018 | 13 |
| 25 April 2020 | Science Direct | mobile health OR mHealth applications OR mHealth apps AND disease diagnosis AND diagnostic accuracy AND treatment support AND health workers AND sub-Saharan Africa | 34,214 | 16 |
