# Supplementary material for: Mapping evidence of mobile health technologies for disease diagnosis and treatment support by health workers in sub-Saharan Africa: a scoping review
Source: BMC Med Inform Decis Mak. 2021 Jan 6;21:11. doi: 10.1186/s12911-020-01381-x (PMC7789784; doi:10.1186/s12911-020-01381-x)
Supplement: Supplementary file 2 — Additional file 2: Full articles screening results and output of degree of agreement in Stata version 13. [file 12911_2020_1381_MOESM2_ESM.docx]

**Additional file 2:** Full articles screening results and output of degree of agreement in Stata version 13

**Yes =1 No =0**

| **Author and date** | **Reviewer 1** | **Reviewer 2** |
| --- | --- | --- |
| Adetunji, 2017 | 0 | 0 |
| Aikens, 2014 | 0 | 0 |
| Akter, 2013 | 0 | 0 |
| Al Dahdah, 2015 | 0 | 0 |
| Alepis ,2013 | 0 | 1 |
| Allard ,2014 | 0 | 0 |
| Aneni, 2013 | 0 | 0 |
| Anstey, 2018 | 0 | 0 |
| Arroz, 2019 | 0 | 0 |
| Atnafu, 2017 | 0 | 0 |
| Babirye, 2019 | 1 | 0 |
| Bassett, 2014 | 0 | 0 |
| Birur, 2019 | 0 | 1 |
| Bitsaki, 2017 | 0 | 0 |
| Bourouis, 2014 | 0 | 0 |
| Boyer, 2010 | 0 | 0 |
| Brath, 2013 | 0 | 0 |
| Brinkel, 2020 | 0 | 0 |
| Catalina, 2010 | 0 | 0 |
| Chang, 2011 | 0 | 0 |
| Chiang, 2018 | 0 | 0 |
| Chib, 2013 | 0 | 0 |
| Chukwu, 2016 | 0 | 0 |
| Coleman, 2017 | 0 | 1 |
| Coulborn, 2018 | 0 | 0 |
| Crankshaw, 2010 | 0 | 0 |
| Davies, 2019 | 0 | 0 |
| Dietrich, 2018 | 0 | 0 |
| Drake, 2016 | 0 | 0 |
| El-Sappagh, 2019 | 0 | 0 |
| Eskenazi, 2014 | 0 | 0 |
| Ettinger, 2016 | 0 | 1 |
| Frandes, 2015 | 0 | 0 |
| Franke, 2018 | 0 | 0 |
| Gbadamosi, 2018 | 0 | 0 |
| Gebremariam,2020 | 0 | 1 |
| Greenleaf, 2019 | 0 | 0 |
| Grolla, 2012 | 0 | 0 |
| Grossman, 2020 | 0 | 0 |
| Guetiya, 2017 | 0 | 0 |
| Hardy, 2017 | 1 | 1 |
| Henry, 2019 | 0 | 0 |
| Henwood, 2016 | 0 | 0 |
| Hermans,2017 | 1 | 0 |
| Hirsch-Moverman, 2017 | 1 | 1 |
| Hwabamungu, 2010 | 0 | 0 |
| Iwu, 2020 | 0 | 0 |
| Jonas, 2016 | 0 | 0 |
| Jonassaint, 2015 | 0 | 0 |
| Kabanda, 2019 | 0 | 0 |
| Kalem, 2015 | 0 | 0 |
| Kamsu, 2014 | 0 | 0 |
| Kaunda, 2018 | 1 | 1 |
| Kawakyu, 2019 | 0 | 0 |
| Kenny,2017 | 0 | 0 |
| Kenny G, 2017 | 0 | 0 |
| Kerber, 2016 | 0 | 0 |
| Kiwanuka, 2018 | 0 | 0 |
| Knight, 2014 | 0 | 0 |
| Korpershoek, 2018 | 0 | 0 |
| Laar, 2019 | 0 | 0 |
| Laidlaw, 2017 | 0 | 0 |
| Lahti, 2017 | 0 | 0 |
| Lamptey, 2017 | 0 | 0 |
| Larissa, 2013 | 1 | 1 |
| Larocca, 2016 | 0 | 0 |
| Leon Natalie, 2017 | 0 | 0 |
| Lester, 2019 | 0 | 0 |
| Lester, 2020 | 1 | 1 |
| Lindgren, 2011 | 0 | 0 |
| Makubi, 2016 | 0 | 0 |
| Marable, 2018 | 0 | 0 |
| Marufu, 2017 | 1 | 1 |
| McNabb, 2015 | 0 | 0 |
| Medhanyie, 2017 | 0 | 0 |
| Mieras, 2018 | 1 | 0 |
| Modena, 2018 | 0 | 0 |
| Modrek, 2014 | 0 | 0 |
| Moodley, 2019 | 1 | 1 |
| Mtema, 2016 | 0 | 0 |
| Nelissen, 2018 | 1 | 1 |
| Neupane, 2014 | 0 | 0 |
| Nhavoto, 2017 | 1 | 1 |
| Nsanzimana, 2012 | 0 | 0 |
| Ochalek, 2018 | 0 | 0 |
| O’Connor, 2015 | 0 | 0 |
| Owolabi, 2020 | 0 | 0 |
| Oyetunde, 2019 | 0 | 0 |
| Pop-Eleches, 2011 | 0 | 0 |
| Potgieter, 2015 | 0 | 0 |
| Roberts, 2015 | 0 | 0 |
| Roeslera, 2015 | 0 | 0 |
| Rono, 2018 | 0 | 0 |
| Ruton, 2018 | 0 | 0 |
| Shao, 2015 | 0 | 0 |
| Sharma, 2019 | 0 | 0 |
| Smillie, 2014 | 1 | 1 |
| Surka, 2014 | 0 | 0 |
| Sutcliffe, 2017 | 0 | 0 |
| Tegegne, 2018 | 0 | 0 |
| van Heerden, 2013 | 0 | 0 |
| van Heerden, 2017 | 0 | 0 |
| van Rooyen, 2013 | 0 | 1 |
| Vasudevan, 2020 | 0 | 0 |
| Vogel, 2016 | 0 | 0 |
| Willcox, 2019 | 0 | 0 |
| Williams, 2019 | 0 | 0 |
| Yahya, 2019 | 1 | 1 |
| Zurovac, 2011 | 1 | 1 |

**Stata output**

Expected

Agreement Kappa Std. Err. Z Prob>Z

-----------------------------------------------------------------

91.74% 74.27% 0.6791 0.0952 7.13 0.0000

| Controls |

Cases | Exposed Unexposed | Total

-----------------+------------------------+------------

Exposed | 12 3 | 15

Unexposed | 6 88 | 94

-----------------+------------------------+------------

Total | 18 91 | 109

McNemar's chi2(1) = 1.00 Prob > chi2 = 0.3173

Exact McNemar significance probability = 0.5078

Proportion with factor

Cases .1376147

Controls .1651376 [95% Conf. Interval]

--------- --------------------

difference -.0275229 -.0903932 .0353473

ratio .8333333 .5826548 1.191863

rel. diff. -.032967 -.0986377 .0327036

odds ratio .5 .0809112 2.341184 (exact)
